# Knockdown of Kremen2 Inhibits Tumor Growth and Migration in Gastric Cancer

**DOI:** 10.3389/fonc.2020.534095

**Published:** 2021-01-07

**Authors:** Beibei Chen, Sai-Qi Wang, Jinxi Huang, Weifeng Xu, Huifang Lv, Caiyun Nie, Jianzheng Wang, Huichen Zhao, Yingjun Liu, Jitian Li, Canrong Lu, Jianying Zhang, Xiao-Bing Chen

**Affiliations:** ^1^ Department of Medical Oncology, Affiliated Cancer Hospital of Zhengzhou University, Henan Cancer Hospital, Zhengzhou, China; ^2^ State Key Laboratory of Esophageal Cancer Prevention & Treatment, Zhengzhou University, Zhengzhou, China; ^3^ Department of Gastrointestinal Surgery, Affiliated Cancer Hospital of Zhengzhou University, Henan Cancer Hospital, Zhengzhou, China; ^4^ Department of Biological Sciences, University of Texas, El Paso, TX, United States; ^5^ Department of General surgery, Chinese People’s Liberation Army General Hospital, Beijing, China

**Keywords:** Kremen2, pathological grade, tumorigenesis, migration, gastric cancer

## Abstract

Kremen2 (Krm2) plays an important role in embryonic development, bone formation, and tumorigenesis as a crucial regulator of classical Wnt/*β*-catenin signaling pathway. However, the role of Krm2 in gastric cancer is not clear. The aim of this study was to explore the regulatory role of Krm2 in the tumorigenesis and metastasis of gastric cancer. It was demonstrated that, compared to para-cancerous tissues, Krm2 was significantly up-regulated in gastric cancer tissues and was positively correlated with the pathological grade of gastric cancer patients. Given that Krm2 is abundantly expressed in most tested gastric cancer cell lines, Krm2 knockdown cell models were established and further used to construct mice xenograft model. After knocking down Krm2, both the cell survival *in vitro* and tumorigenesis *in vivo* of gastric cancer cells were inhibited. At the same time, knockdown of Krm2 induced apoptosis, cell cycle arrest at G_2_/M phase and repression of migration in gastric cancer cells *in vitro*. Mechanistically, we found that knockdown of Krm2 suppressed PI3K/Akt pathway. Therefore, we revealed the novel role and the molecular mechanism of Krm2 in promoting the tumorigenesis and metastasis in gastric cancer. Krm2 can be a potent candidate for designing of targeted therapy.

## Introduction

Gastric cancer (GC) is the third leading cause of cancer-related death in the world (1), and about 50% gastric cancer occurs in China. Until now, the only treatment that offers a potential cure is complete surgical resection. However, since the disease is asymptomatic in its early stages, more than half of patients are diagnosed in the advanced stage of tumor, for whom resection is no longer possible. Therefore, chemotherapy is another major method for treating gastric cancer. However, for patients with advanced gastric cancer, traditional cytotoxic chemotherapy only results in a median survival of 9–11 months (2), and the 5-year survival rate of metastatic gastric cancer is less than 10%. In recent years, molecular targeted therapy has brought new hope to the therapy of gastric cancer. Therefore, it is necessary to deepen the understanding of the molecular mechanism of gastric carcinogenesis and identify potential drug-targets for GC targeted therapy.

Kremen is a kind of protein containing Kringle domain which was originally identified in the blood coagulation factor prothrombin, as repeating homologous triple-disulfide-linked peptide regions (3). Mammalian Kremen1 (Krm1) is a kind of dependence receptors regulating cell death pathways to remove abnormal or unwanted cells, and Krm2 is a very potent inhibitor through blocking homodimerization of Krm1. In addition, Kremen functions as receptors of Dickkopf1 (Dkk1) to regulate Wnt/*β*-catenin pathway (4–6). Cselenyi and Lee found that “Binding of a Wnt to its transmembrane co-receptors Frizzled (Fz) and low-density lipoprotein (LDL) receptor-related protein (LRP) 5 or LRP6 inhibits the degradation of the transcriptional coactivator *β*-catenin, which translocates to the nucleus to regulate gene expression. The secreted protein Dkk1inhibits Wnt signaling by binding to LRP5 and LRP6 and blocking their interaction with Wnt and Fz. Krm1 and 2 (Krm1 and 2, collectively termed Krms) are single-pass transmembrane Dkk1 receptors that synergize with Dkk1 to inhibit Wnt signaling by promoting the endocytosis of LRP5 and LRP6” (7–9).

Mass studies have shown that Krm2 may be involved in embryonic development, bone formation, neural ridge formation and tumorigenesis (10, 11). Data from The Cancer Genome Atlas (TCGA) show that Krm2 expression was higher in tumor compared with normal tissue in >80% of samples considered, regardless of the cancer types. Especially, in lung squamous cells carcinoma, the median increase is larger than 10-fold (5). However, the expression level and role of Krm2 in gastric cancer are still not clear. The purpose of this study is to explore the expression level of Krm2 in gastric cancer and its relationship with patients’ pathological features to clarify the regulatory role of Krm2 in the development and metastasis of gastric cancer and to lay a foundation for the development of new therapeutic targets for gastric cancer.

## Materials and Methods

### Specimens and Tissue Microarray

Total 208 specimens including 156 gastric adenocarcinomas, 40 gastric adenocarcinomas with distant metastasis, eight adjacent normal stomach tissues and four normal stomach tissues were made into tissue microarray in this study. Briefly, formalin-fixed, paraffin-embedded specimens were mapped by a pathologist for coring. The tissue microarray was constructed with 1.0-mm diameter cores spaced 0.8 mm apart using a modified Tissue Microarrayer (Beecher Instruments, Sun Prairie, WI, USA). All gastric cancer patients were pathologically diagnosed after surgical operations. All the pathologic data were provided in **Supplementary Table 1**. All subjects gave written informed consent in accordance with the Declaration of Helsinki. The experimental processes were performed with the approval of Medical Ethics Committee of the Henan Cancer Hospital.

### Cell Culture

Human gastric carcinoma cell lines (BGC-823 and SGC-7901) were cultured in RPMI 1640 containing 10% fetal bovine serum (Biological Industries, Kibbutz Beit Haemek, Israel), 100 units/ml penicillin and 100 μg/ml streptomycin with 5% CO2 at 37°C.

### Real-Time Quantitative Polymerase Chain Reaction

qRT-PCR was performed with a standard SYBR Green PCR Kit (Vazyme, China) on an VII7 Real-Time PCR system (Applied Biosystems, Waltham, MA). Glyceraldehyde 3-phosphate dehydrogenase (GAPDH) was used for normalization. Primers for Krm2: Forward: 5′-GCGCACAACTTCTGCCGTAAC-3′; Reverse: 5′-GTGCCCCTGAGTCCACAAAGC-3′. Primers for GAPDH: Forward: 5′-TGACTTCAACAGCGACACCCA-3′; 5′-CACCCTGTTGCTGTAGCCAAA-3′.

### Construction of the Lentivirus and Transfection

Lentiviral constructs repressing *KREMEN2* (target sequence: CCTACGCTTCTGCCGCATGAA) purchased from GenePharma (Suzhou, China) were used to knockdown *KREMEN2*. The fragment with the highest efficiency verified by qRT-PCR and western blot was selected from three interference fragments of Krm2.

### Cell Viability Detection

Cell viability was determined by the MTT assay according to the manufacturer’s instructions. Briefly, 20 μl of MTT was added to each well at a final concentration of 500 μg/ml, and the plates were incubated for 4 h at 37°C. After staining, the supernatant was removed, and 150 μl of dimethyl sulfoxide (DMSO) was added to each well followed by vigorous shaking; the absorbance was determined at 570 nm. The cell viability assay was performed on days 1, 2, 3, 4, and 5. The percent of cytotoxicity was calculated using the following formula: %Cell viability = (absorbance of experimental well-absorbance of blank)/(absorbance of untreated control well-absorbance of blank)×100% (12).

### Apoptosis Assay

Cell apoptosis was detected using the eBioscience™ Annexin V Apoptosis Detection Kit APC (Invitrogen, Carlsbad, CA) according to the manufacturer’s instructions. In brief, prepared cells were washed twice with cold PBS and resuspended gently in 500 μl binding buffer containing 5 μl fluorochrome-conjugated Annexin V staining solution. 5 min later, cell samples were detected by Guava easyCyte HT flow cytometer (Millipore Corp., Billerica, MA).

### Cell Cycle Distribution

Cell cycle distribution was detected through analysis of DNA content using flow cytometry. Briefly, cells were collected and fixed with ice-cold ethanol (70%). The cells were then treated with buffer containing RNaseA and 0.1% Triton X-100 and then stained with PI (Sigma, St. Louis, MO). DNA content was detected on FACSCalibur flow cytometer (Becton Dickinson, Franklin Lakes, NJ). Data was analyzed using the FlowJo software (Tree Star, Inc., Ashland, OR, USA).

### Wound Healing and Migration Assay

Cells were seeded onto 6-well plates. The monolayer was scratched with a 10 μl pipette tip and then incubated in serum-free RPMI-1640 medium. Images of the wells were taken at different time points (0, 24, and 48 h) on an inverted microscope (Axioskop 2 Plus; Zeiss, Germany). The length of the open area was calculated with software. Cell migration was evaluated using Transwell inserts with 8 μm pores (BD Biosciences, San Jose, CA, USA). Briefly, cells were released using trypsin-EDTA and sequentially rinsed with RPMI-1640 medium containing 10% FBS. The rinsed cells were resuspended in serum-free RPMI-1640 medium, and 200 μl of the cell suspension (1 × 10^5^ cells) was added to the Transwell insert chamber with a filter that was coated with Matrigel. In the lower chamber, 500 μl of RPMI-1640 medium containing 10% FBS was added as a chemoattractant. After 24 h of incubation, the cells in the lower chamber were fixed, stained with hematoxylin and counted under a microscope.

### Immunohistochemistry

The IHC analysis comprised 196 gastric cancer tissues, eight adjacent normal stomach tissue and four stomach tissues. Briefly, tissue microarray was retrieved with citrate buffer and incubated with a Krm2 primary antibody (1:100, Abcam, England, # ab156007). The protein expression was detected by DAB horseradish peroxidase color development kit (Beyotime, Haimen, China). The results of staining in the sections was observed and scored independently by two pathologists according to the following criteria: (1) The immunostaining intensity: none: 0, weak (I): 1, moderate (II): 2, strong (III): 3; (2) The positive expression rate (%): none: 0, below 25%: 1, 25−49%: 2, 50−74%: 3, above 75%: 4. The expression score of Krm2 in each specimen was calculated as follows: expression score = (1) × (2). The expression scores of Krm2 were provided in **Supplementary Table 2**.

### Western Blot Analysis

Protein lysates were separated using 10% SDS-PAGE and transferred onto a PVDF membrane (Roche, Switzerland). Then, membranes were incubated with antibodies against Krm2 (1:1,000, Abcam, Cambridge, MA), Akt (1:1,000, Cell Signaling Technology, Danvers, MA), p-Akt (1:1,000, Bioss, China), E2F1 (1:1,000, Abcam, Cambridge, MA), Cyclin D1 (1:2,000, Cell Signaling Technology, Danvers, MA) or PIK3CA (1:1,000, Abcam, Cambridge, MA) followed by incubation with secondary antibodies (1:3,000, Beyotime, China). Finally, the proteins were detected using an ECL-PLUS/Kit detection system. The GAPDH (1:3,000, Bioworld, China) was used as the loading control.

### *In Vivo* Antitumor Activity and *In Vivo* Imaging

All experimental procedures were approved by the Zhengzhou University ethical committee. To evaluate tumor growth *in vivo*, xenograft tumors were generated by subcutaneously injecting 4 × 10^6^ the luciferase-expressing cells using 4-week-old male BALB/c nude mice (Beijing Vital river, China). The volume of tumors was measured as follows: tumor volume (mm^3^) = length × width × width/2. The tumors were fixed in 4% paraformaldehyde and were embedded in paraffin for Ki67 detection using IHC.

For *in vivo* imaging, bioluminescence was observed at the end of the experiment *via* an *in vivo* imaging system (IVIS Spectrum, Perkin Elmer, Hopkinton, MA). Briefly, after intraperitoneal injection of D-Luciferin (150 mg/kg, Shanghai Qianchen Biological Technology, China), the animals were anesthetized with 2% isoflurane for *in vivo* imaging.

### Statistical Analysis

Each experiment was repeated at least three times, and data were presented as means ± S. D. Statistical significance between two groups was evaluated using Student’s t-test. Mann–Whitney U test was applied to compare the pathological parameters. The relationship between Krm2 expression and pathologic data was determined with Pearson’s correlation analysis. All statistical analyses were performed with SPSS 22.0 software (SPSS, Inc., Chicago, USA). *P <*0.05 was regarded as significant. Data were graphed using GraphPad Prism 8 (GraphPad Software, San Diego, CA).

## Results

### Krm2 Was Highly Expressed in GC Tissues and Cells and Was Positively Correlated With Pathological Grade

**Figure 1:**
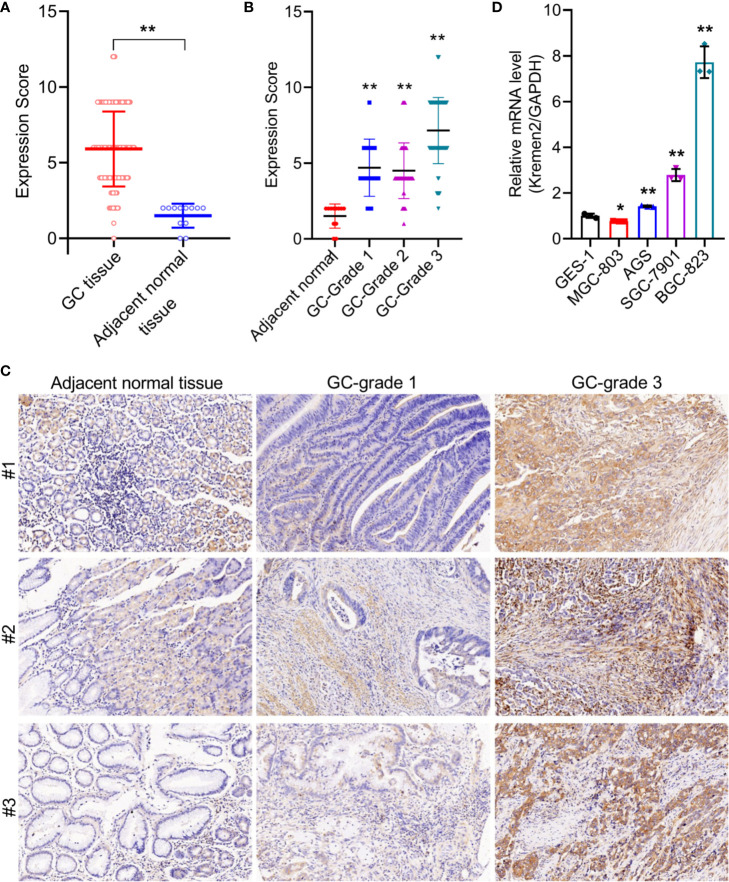
Kremen2 was highly expressed in GC tissues and cultured cell lines, and was positively correlated with pathological grade. **(A)** The protein levels of Kremen2 in gastric cancer tissues and adjacent normal tissues. **(B)** The protein levels of Kremen2 adjacent normal and cancer with different pathological grade. Kremen2 expression of 196 gastric cancer tissues, eight adjacent normal stomach tissue and four stomach tissues were detected using IHC, and expression score was calculated according to immunostaining intensity and positive expression rate. **(C)** Kremen2 expression was positively correlated with pathological grade. **(D)** Kremen2 expression was up-regulated AGS, SGC-7901, BGC-823, while down-regulated in MGC-803 gastric cancer cell line, compared with normal gastric mucosa epithelial cell line GES-1. The mRNA levels of Kremen2 were measured by qRT-PCR. *P < 0.05; **P < 0.01; ***P < 0.001.

To verify the expression of Krm2 in GC tumor tissues and cells, IHC and qRT-PCR were performed on collected samples from patients and cultured cells, respectively. As shown in **Figure 1A**, outcomes of IHC analysis revealed that Krm2 was significantly up-regulated in GC tissues compared with adjacent normal tissues. The expression level of Krm2 in GC tissues was higher than that of in para-carcinoma tissues. Based on the median value of Krm2 expression, the tissues were divided into high Krm2 expression group (n = 121) and low Krm2 expression group (n = 75). Obviously, 61.7% (121/196) of them exhibited relatively high expression of Krm2 while all the para-carcinoma tissues exhibited low expression (*P* < 0.001, **Table 1**). The relationship between Krm2 protein levels and the clinical features of the clinicopathologic data of GC patients was statistically analyzed and presented in **Table 2**. Spearman correlation analysis also showed that Krm2 gene expression was positively correlated with pathological grade (**Table 3**). Taken together, with the increase of tumor malignancy, Krm2 gene expression increased (**Table 3** and **Figures 1B, C**). Furthermore, results of qRT-PCR showed that, compared with normal gastric mucosa epithelial cell line (GES-1), Krm2 expression was up-regulated in AGS (1.42-fold), SGC-7901 (2.79-flod), BGC-823 (7.72-fold), while down-regulated in MGC-803 (76.5%), (**Figure 1D**).

**Table 1:** Expression patterns in gastric cancer tissues and adjacent normal tissues revealed in immunohistochemistry analysis.

Kremen2 expression	Tumor tissue		Para-carcinoma tissue		p value
	Cases	Percentage	Cases	Percentage	
Low	75	38.3%	12	100%	<0.001
High	121	61.7%	0	/	

**Table 2:** Relationship between Kremen2 expression and tumor characteristics in patients with gastric cancer.

Features	No. of patients	Kremen2 expression		p value
		low	high	
All patients	196	75	121	
Age (years)				0.255
≤56	99	34	65	
>56	97	41	56	
Gender				0.091
Male	135	57	78	
Female	61	18	43	
Grade				0.000**
1	13	8	5	
2	56	41	15	
3	106	12	94	
Stage				0.717
I	24	8	16	
II	79	33	46	
III	39	11	28	
IV	14	6	8	
T Infiltrate				0.343
T1	4	1	3	
T2	25	7	18	
T3	116	46	70	
T4	11	4	7	
Lymphatic metastasis (N)				0.558
N0	104	41	63	
N1	34	9	25	
N2	12	6	6	
N3	6	2	4	

**P < 0.01.

**Table 3:** Relationship between Kremen2 expression and tumor characteristics in patients with gastric cancer.

		Krm2
Grade	Pearson Correlation	0.589
	Significant (Double tail)	<0.001
	N	175

### Krm2 Knockdown Suppressed the Survival of GC Cells *In Vitro*

**Figure 2:**
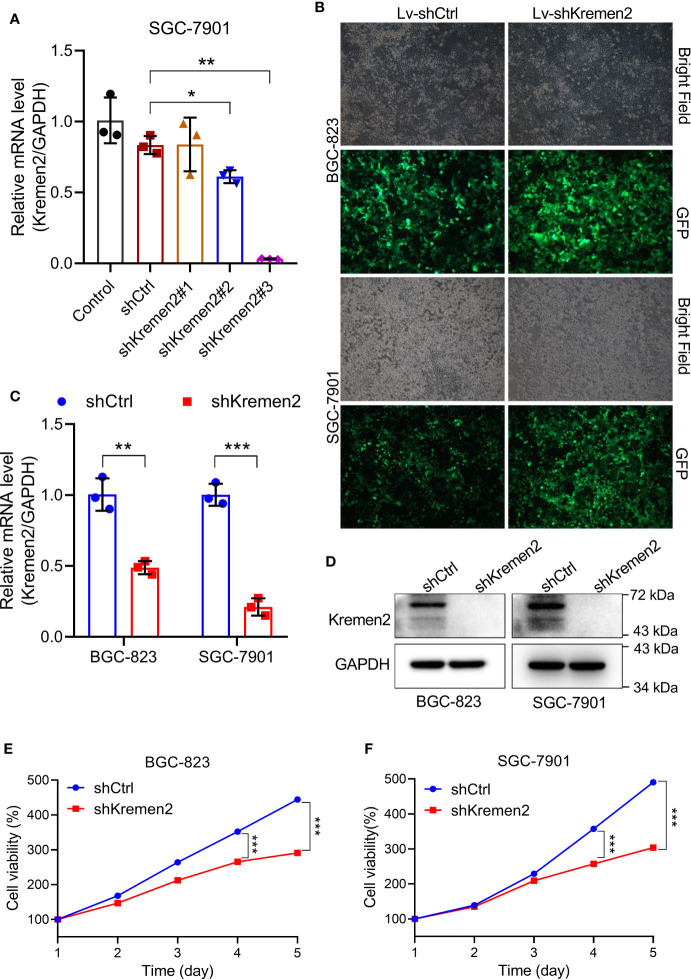
Kremen2 knockdown suppressed cell survival of GC *in vitro*. **(A)** SGC-7901 cells were transfected with Lenti-shKremen2 (#1, #2 and #3) or Lenti-shCtrl, and the mRNA level of Kremen2 was measured by qRT-PCR. **(B)** Infection efficiency of Lenti-shKremen2_#3 was above 80%. **(C)** mRNA levels of Kremen2 in BGC-823 and SGC-7901 cells after transfection with Lenti-shKremen2. **(D)** Protein levels of Kremen2 in BGC-823 and SGC-7901 cells after transfection with Lenti-shKremen2. **(E)** BGC-823 cell survival after Kremen2 knockdown was detected using MTT assay. **(F)** SGC-7901 cell survival after Kremen2 knockdown was detected using MTT assay. *P < 0.05; **P < 0.01; ***P < 0.001.

To explore the biological relevance of Krm2 in gastric cancer, loss-of-function experiments were performed. To screen the best shRNA candidate for knocking down Krm2, SGC-7901 cells were infected with lentivirus expressing different shRNAs or shCtrl. The results of qRT-PCR showed that the knockdown-efficiency of shKrm2#1, #2 and #3 was −0.6, 26.7, and 96.2%, respectively (**Figure 2A**). Therefore, shKrm2#3 was used in subsequent experiments for silencing Krm2. Subsequently, BGC-823 and SGC-7901 cells were infected with shCtrl or shKrm2 for 72 h, a >80% efficiency of which was observed by fluorescence microscope (**Figure 2B**). The results of qRT-PCR showed that the knockdown efficiency of Krm2 in BGC-823 and SGC-7901 cells, was 51.4% (*P* < 0.01) and 78.9% (*P* < 0.001), respectively (**Figure 2C**). In addition, the results of Western blot showed that, compared with shCtrl group, the protein level of Krm2 in shKrm2 group is down-regulated (**Figure 2D**). Next, the effects of Krm2 knockdown on gastric cancer cell viability were assessed using MTT assay. As shown in **Figures 2E, F**, after Krm2 knockdown, the survival of BGC-823 and SGC-7901 cells was inhibited.

### Krm2 Knockdown Induced Apoptosis and Cell Cycle Arrest at G_2_/M in Gastric Cancer Cells

**Figure 3:**
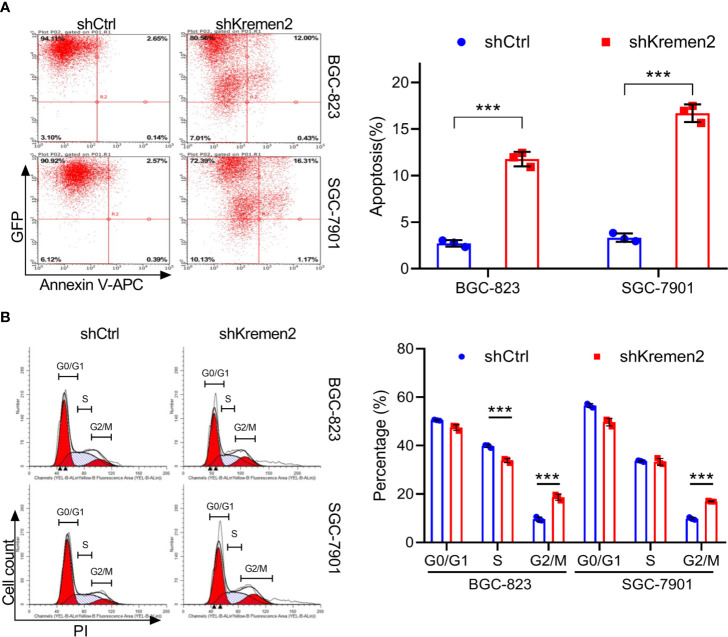
Kremen2 knockdown induced apoptosis and cell cycle arrest at G2/M phase in GC cells. GC cells transfected with Lenti-shKremen2 or Lenti-shCtrl. **(A)** Kremen2 knockdown triggered apoptosis of GC cells. Transfected GC cells were stained with Annexin V-APC to detect by flow cytometry. **(B)** Kremen2 knockdown induced cell arrest of GC cells at G2/M phase. Transfected GC cells were fixed and stained with PI to analyze DNA content by flow cytometry. ***P < 0.001.

To examine whether Krm2 knockdown affects apoptosis, we performed flow cytometry analysis by staining cells with Annexin V-APC. As seen in **Figure 3A**, for BGC-823 cells, the apoptosis rate was (2.72 ± 0.35%) in the control group and was (11.70 ± 0.78%) in the shKrm2 group. Similarly, in SGC-7901 cells, the apoptosis rate was (3.33 ± 0.45%) and (16.69 ± 0.95%) in cells without or with Krm2 knockdown. These data revealed that Krm2 knockdown induced apoptosis in GC cells. To explore the role of Krm2 in cell cycle distribution, we performed flow cytometry analysis by staining cells with propidium iodide. As can be seen in **Figure 3B**, in BGC-823 cells, the percentage of G_2_/M phase of control group was (9.64 ± 0.88%), which went up to (18.7 ± 1.28%) in the shKrm2 group. At the same time, the percentage of G0/G1 and S-phase cells decreased. A similar phenomenon could be observed in SGC-7901 cells. After KREMEM2 knockdown, the percentage of G_2_/M phase cells increased from (9.84 ± 0.51%) to (16.98 ± 0.19%). These data revealed that Krm2 knockdown induced G_2_/M phase arrest in gastric cancer cells.

### Krm2 Knockdown Inhibited Migratory Ability in Gastric Cancer Cells

**Figure 4:**
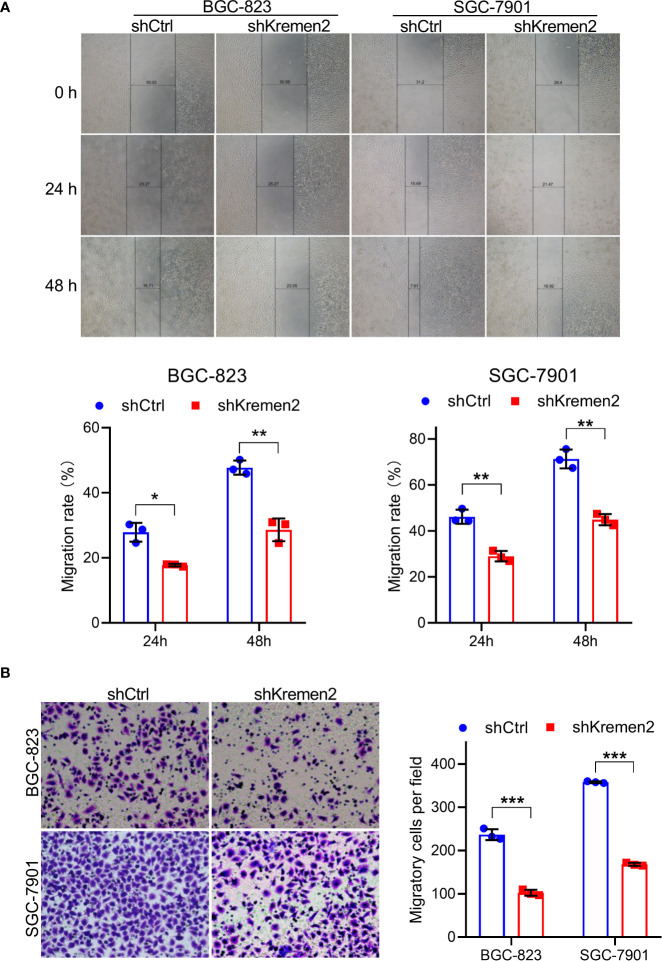
Kremen2 knockdown inhibited migration of GC cells. **(A)** Kremen2 knockdown blocked wound healing of GC cells. The width was measured at 0, 24, and 48 h after scratching. **(B)** Kremen2 knockdown inhibited migration of GC cells. Transfected GC cells were cultured in Transwell insert chamber, 24 h later, the cells in the lower chamber were fixed, stained with hematoxylin. *P < 0.05; **P < 0.01; ***P < 0.001.

In order to further investigate the role of Krm2 in metastasis, wound healing and Transwell assays were performed. As shown in **Figure 4A**, in the control group, the migration rate of BGC-823 cells was (28 ± 3%) and (48 ± 2%) at 24 and 48 h, while after knockdown of Krm2, the migration rate was (18 ± 0.1%) and (29 ± 4%) at 24 and 48 h. Likewise, in SGC-7901 cells, knockdown of Krm2 reduced migration rate from (46 ± 3%) to (29 ± 2%) at 24 h, and (71 ± 4%) to (45 ± 2%) at 48 h. In addition, the results of Transwell assay also illustrated that knockdown of Krm2 significantly reduced migration of BGC-823 and SGC-7901 cells (**Figure 4B**). These data suggested that Krm2 knockdown inhibited migratory ability of gastric cancer cells *in vitro*.

### Krm2 Knockdown Inhibited Proliferation of Gastric Cancer Cells Through the PI3K/Akt Pathway

To explore the mechanisms by which Krm2 regulates gastric cancer progression, we first detected the apoptosis-related protein using Human Apoptosis Antibody Array.

**Figure 5:**
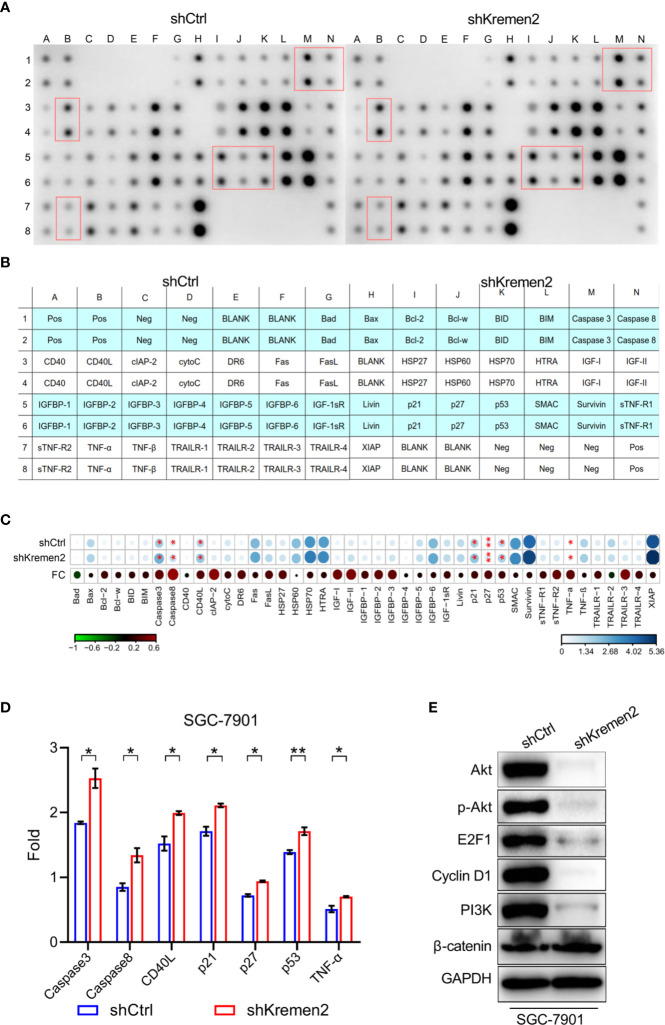
Kremen2 knockdown inhibited survival of GC cells through the PI3K/Akt pathway. SGC-7901 cells were stably transfected with Lenti-shKremen2 or Lenti-shCtrl and cell lysate was analyzed using antibody array and western blot. **(A)** The spots of apoptosis antibody array after knockdown of Kremen2. **(B)** The position of each protein in the antibody array. **(C)** Fold change of each protein in the antibody array. **(D)** Semi-quantitative analysis of change of caspase 3, caspase 8, CD40L, p21, p27, p53 and TNF-α following Kremen2 knockdown. **(E)** Kremen2 knockdown down-regulated expression of p-Akt, Akt, E2F1, PI3K and cyclin D. *P < 0.05; **P < 0.01.

The actual spots of antibody array were shown in **Figure 5A**, in which and the red boxes highlighted the proteins significant up-regulated after knockdown of Krm2 (*P* < 0.05). The position of each protein in the antibody array was shown in **Figure 5B**. The fold change (FC) of each protein was shown in **Figure 5C** with an asterisk indicating *P* < 0.05. During the 43 tested proteins, caspase 3, caspase 8, CD40L, p21, p27, p53, and TNF-α were significantly up-regulated after knockdown of Kremen2 (**Figure 5D**). These results suggested that the inhibited cell proliferation by Krm2 knockdown may be related to regulation of apoptosis and cell cycle arrest, so we next tested the changes of proteins in PI3K/Akt pathway. We found that, after Krm2 knockdown, expression levels of p-Akt, Akt, E2F1, PI3K, and cyclin D1 sharply decreased in SGC-7901 cells. In addition, no obvious change of *β*-catenin was observed after knockdown of Krm2 (**Figure 5E**). These data showed that Krm2 knockdown inhibited growth of gastric cancer cells by restraining PI3K/Akt pathway.

### Krm2 Knockdown Repressed Tumorigenicity of Gastric Cancer Cells *In Vivo*

**Figure 6:**
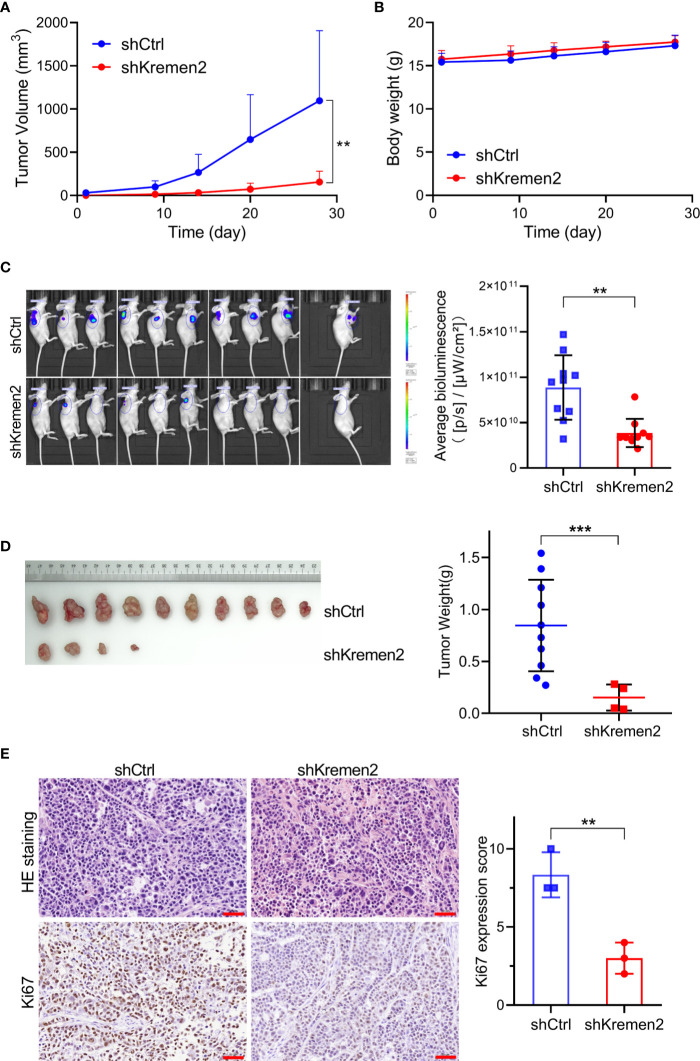
Kremen2 knockdown repressed tumorigenicity of gastric cancer cells *in vivo.* Transfected GC cells expressing luciferase were subcutaneously injected to establish xenografts. Data including tumor volume and bodyweight were collected every two days. At the end of the *in vivo* study, bioluminescence was observed *via* an *in vivo* imaging system. **(A)** Tumor volume. **(B)** Body weight of nude mice. **(C)** *In vivo* imaging weight of tumor at the end of experiment (Day 28). **(D)** Tumors and tumor weight at the end of experiment. **(E)** H&E and Ki67 analysis of paraffin-embedded tumor tissues, and Ki67 expression score was calculated. **P < 0.01; ***P < 0.001. Scale bar is 50 μm.

Subsequently, we tested the role of Krm2 knockdown on tumorigenicity of gastric cancer *in vivo* through establishing mice gastric cancer xenografts. Immunodeficient Balb/C mice were subcutaneously injected with SGC-7901 cells stably transfected with Lenti-shKrm2 or Lenti-shCtrl. Throughout the tumorigenic period, the tumors that generated from Lenti-shKrm2 stably transfected SGC-7901 cells grew significantly slower than those generated from Lenti-shCtrl transfected SGC-7901 cells (**Figure 6A**), while there was no significant difference in body weight between the two groups (**Figure 6B**). At the last day of the experiment, tumor size was measured using *in vivo* imaging (**Figure 6C**). Then, tumors were taken out and weighed, showing that tumor weight of the control group and Krm2 knockdown group was (0.85 ± 0.44) g and (0.15 ± 0.12) g, respectively (**Figure 6D**). Subsequently, immunohistochemical (IHC) analysis of tumors indicated that Ki67 expression was significantly lower in the Krm2 knockdown group (**Figure 6E**). These data suggested that Krm2 may contribute to tumor growth of gastric cancer cell *in vivo*.

## Discussion

Krm2 has been found to play important roles in development (11), bone formation (13) and tumorigenesis (5, 14–16). As an inhibitor of Krm1, Krm2 inhibits cell apoptosis by inhibiting homodimerization of Krm1. On the other hand, Krm2 acts as a receptor of Dkk1 to form a trimer with LRP5/6 protein to internalize LRP5/6, blocking the formation of Wnt-LRP5/6-Frizzled complex, and enhances the Dkk1-mediated inhibition of Wnt/*β*-catenin signaling pathway. However, another study found that in the absence of Dkk1 protein, Krm2 can bind to LRP5/6 and maintain its presence on cell membrane, thus activating Wnt/*β*-catenin signaling pathway (9). Therefore, it can be inferred that Krm2 has different biological effects in different biological environments.

Recent studies show that, compared with normal tissue, Krm2 is up-regulated in several kinds of tumor including breast invasive carcinoma, colon adenocarcinoma, stomach adenocarcinoma (5). Combined Dkk1 and Krm expression in cancer cells may serve as predictive markers of the osteoblastic response of breast and prostate cancer bone metastasis (17). However, the expression and role of Krm2 in gastric cancer remain elusive. In this study, we found that Krm2 is significantly up-regulated in gastric cancer, which may be involved in the carcinogenesis and metastasis of gastric cancer.

First, we found that the protein level of Krm2 in gastric cancer tissues and most gastric cancer cell line was significantly higher than that in para-cancerous tissues. This result is consistent with previous reports (5). Although no correlation between the level of Krm2 and the age, sex, or TNM stage could be observed, we found that the expression of Krm2 gene was positively correlated with the pathological grade according to the Spearman grade correlation analysis. Therefore, Krm2 expression may be associated with the progression of gastric cancer. In the future, we will observe the survival of these gastric cancer patients, and explore the relationship between Krm2 and the overall survival of gastric cancer patients.

As is well known, Krm1 is a dependence receptor, triggering cell death unless bound to its ligand Dkk1 or Krm2 in a Wnt-independent manner (4). We speculated that inhibition of Krm2 may induce cell death. After knocking down Krm2, we found that, the proliferation of gastric cancer cells was significantly inhibited, suggesting that Krm2 may possess oncogene-like functions to promote the proliferation of gastric cancer. Next, we explored the molecular mechanism of Krm2 to promote gastric cancer development. We found that knockdown of Krm2 triggered apoptosis and cell cycle arrested at G_2_/M phase in gastric cancer cells. These results suggested that Krm2 may be involved in the regulation of apoptosis and cell cycle in gastric cancer. Therefore, we used Human Apoptosis Antibody Array to detect the changes of apoptosis and cell cycle-related proteins after Krm2 knockdown. The results showed that the expression of caspase 3, caspase 8, p53, p21, p27, and TNF-α was significantly up-regulated after Krm2 knockdown. Further experiments showed that Krm2 knockdown inhibited the expression of PI3K, p-Akt, Cyclin D1 and E2F1. As we know, caspase 3 and caspase 8 mediate external apoptotic pathways (18), so we speculated that Krm2 knockdown may lead to apoptosis through death receptor pathway. p53 is a tumor suppressor gene, which is degraded by its E3 ubiquitin ligase MDM2 to maintain low level in cytoplasm. p21 is a member of the ClP family. It is a cyclin-dependent kinase inhibitor and is a downstream target of p53 (19). After DNA damage without repair, cells cannot pass the G1 checkpoint formed by p21 and p53, replication and accumulation of damaged DNA was suppressed (20). p27 mainly binds to cyclin to inhibit cyclin-CDK complex to block cell cycle progression and cell division (21). Therefore, we guess that Krm2 promotes cell cycle progression by regulating p21 and p27.

PI3K/Akt is an important signaling pathway that regulates cell proliferation and has been found to be abnormally activated in a variety of tumors. PI3K activates the transcription of its downstream target genes and promotes cell proliferation by phosphorylating Akt (20, 22). As Akt can induce the degradation of p53 through phosphorylation of MDM2 (23), therefore, we speculated that knockdown of Krm2 may inhibit the phosphorylation of Akt by PI3K and de-activates of PI3K/Akt pathway, resulting in up-regulation of p53. Up-regulated p53 activated transcription of its downstream targets p21 and p27, then decreased expression of cyclin D1, leading to cell cycle arrest. At the same time, after Krm2 knockdown, the migration ability of gastric cancer cells also decreased, the mechanism of which is still not clear and will be explored in our future study.

In the *in vivo* experiment, we found that the growth of tumor was significantly inhibited after Krm2 knockdown, and the T/C (%) of the Krm2 knockdown group was 25.2% (*P* < 0.001). The results of Ki67 staining showed that the proliferative activity of tumor cells was suppressed in Krm2 knockdown group.

In summary, we demonstrated that Krm2 was up-regulated in gastric cancer and related to pathological grade. We revealed that Krm2 may promote gastric cancer proliferation and metastasis by activating PI3K/Akt pathway, and Krm2 may be a biomarker of grading and a potential therapeutic target in gastric cancer.

## Data Availability Statement

All datasets generated for this study are included in the article/**Supplementary Material**.

## Ethics Statement

All subjects gave written informed consent in accordance with the Declaration of Helsinki. The experimental processes were performed with the approval of Medical Ethics Committee of the Henan Cancer Hospital.

## Author Contributions

BC conceived and designed the experiments. BC, S-QW, and JH performed the experiments and collected the data. WX, HL, and CN performed statistical analysis. BC and S-QW wrote the first draft of the manuscript. JH, JW, HZ, and YL collected the clinical specimens and pathological data. JL analyzed the data. X-BC designed the experiment, supervised the entire study, and revised the manuscript. JZ and CL revised the manuscript for intellectual content. All authors contributed to the article and approved the submitted version.

## Funding

We would like to thank the financial support from the National Natural Science Foundation of China (No. 81472714), 1000 Talents Program of Central plains (No. 204200510023), Project of international scientific and technological cooperation research of Henan Province (No. 182102410023), Science and Technique Foundation of Henan Province (No. 202102310413), Medical Science and Technique Foundation of Henan Province (Nos. 2018020486 and SB201901101), Young and middle-aged Health and Technology Innovation Leading talent Project of Henan Province (YXKC2020008) and State Key Laboratory of Esophageal Cancer Prevention & Treatment (No. Z2020000X).

## Conflict of Interest

The authors declare that the research was conducted in the absence of any commercial or financial relationships that could be construed as a potential conflict of interest.
